# Limits to sustained energy intake XXIV: impact of suckling behaviour on the body temperatures of lactating female mice

**DOI:** 10.1038/srep25665

**Published:** 2016-05-09

**Authors:** Y. Gamo, A. Bernard, C. Troup, F. Munro, K. Derrer, N. Jeannesson, A. Campbell, H. Gray, J. Miller, J. Dixon, S. E. Mitchell, C. Hambly, L. M. Vaanholt, J. R. Speakman

**Affiliations:** 1 Institute of Biological and Environmental Sciences, University of Aberdeen, Aberdeen, AB24 2TZ, Scotland, UK; 2 Institute of Genetics and Developmental Biology, State Key Laboratory of Molecular Developmental Biology, Chinese Academy of Sciences, Beichen Xi Lu, Chaoyang, Beijing, People’s Republic of China

## Abstract

The objective of this study was to investigate the potential causes of high body temperature (T_b_) during lactation in mice as a putative limit on energy intake. In particular we explored whether or not offspring contributed to heat retention in mothers while suckling. Tb and physical activity were monitored in 26 female MF1 mice using intraperitoneally implanted transmitters. In addition, maternal behaviour was scored each minute for 8 h d^−1^ throughout lactation. Mothers that raised larger litters tended to have higher T_b_ while nursing inside nests (*P* < 0.05), suggesting that nursing offspring may have influenced heat retention. However, T_b_ during nursing was not higher than that recorded during other behaviours. In addition, the highest T_b_ during the observation period was not measured during nursing behaviour. Finally, there was no indication that mothers discontinued suckling because of a progressive rise in their T_b_ while suckling. T_b_ throughout lactation was correlated with daily increases in energy intake. Chronic hyperthermia during lactation was not caused by increased heat retention due to surrounding offspring. Other factors, like metabolic heat produced as a by-product of milk production or energy intake may be more important factors. Heat dissipation limits are probably not a phenomenon restricted to lactation.

Food intake at peak lactation in small mammals reaches an asymptote and appears to be limited^1^. The level of the asymptotic intake is strongly dependent on ambient temperature, with lower ambient temperatures leading to greater food intakes^1^,^2^,^3^,^4^,^5^. On the basis of these observations it has been suggested that hyperthermia risk is a potential factor limiting energy intake in lactating mice (the heat dissipation limit theory)^5^,^6^,^7^,^8^,^9^,^10^. Consistent with this hypothesis shaved female MF1 mice increased their food intake and raised bigger pups at weaning^11^. However, Swiss mice that were shaved during lactation ate more food, but did not significantly increase their milk production or raise heavier pups^12^,^13^, and a similar absence of any impact of shaving was observed in hamsters^14^. Nevertheless, data from larger domesticated animals strongly support the view that hyperthermia risk is a key factor limiting lactation performance^15^,^16^. Moreover, direct observations suggest that lactating MF1 mice are chronically hyperthermic compared to non-reproductive and pregnant individuals^17^,^18^ and this is consistent with more sporadic body temperature (T_b_) measurements reported in several other small rodents during lactation, such as Mongolian gerbils (*Meriones unguiculatus*)^19^, Dwarf hamsters (*Phodopus campbelli* and *P. sungorus*)^20^ and Sprague-Dawley rats (*Rattus norvegicus*)^21^. These data suggest hyperthermia risk may be a broadly applicable limitation on lactation performance.

High T_b_ in lactating mice might be a direct consequence of an increase in food consumption, due to the thermic effect of food (also called the heat increment of feeding or specific dynamic action- which reflects the increase in heat production following food ingestion), as well as heat generated during milk synthesis. An alternative hypothesis for the hyperthermia of lactation is that lactating animals face problems dissipating heat because of the surrounding pups when they are nursing, and also the fact the pups need to be sheltered in a nest. Both the pups and the nest adjacent to the nursing mother may affect her ability to dissipate heat. Supporting this viewpoint, in lactating Norway rats (*Rattus norvegicus*), increases in core T_b_ and ventrum temperature occurred acutely during nursing bouts either under a warm room temperature at 26 °C or with warmed (39 °C) pups^22^. An increase in maternal T_b_ caused rats to terminate nursing bouts^22^,^23^. Warming pups resulted in many short nursing bouts in lactating rats (*Rattus norvegicus*) with unchanged total contact time^22^. Moreover the time spent nursing increased at lower ambient temperatures. On the other hand, other work suggested that rats did not terminate nursing bouts in response to an increase in core T_b_^24^. In addition during approximately 60% of nursing bouts recorded in lactating rats (*Rattus norvegicus*), higher T_b_ were observed during nursing bouts, rather than at the end of the bouts^25^. Therefore, whether or not surrounding pups have a direct effect on maternal T_b_, thereby influencing nursing behaviour remains uncertain. Understanding whether the hyperthermia of lactation is caused mostly by increased heat production or reduced capacity to dissipate heat is important because if it was the former it might point to heat dissipation being a more generally significant factor limiting maximum sustained metabolic rate, as opposed to a lactation specific effect^8^,^10^.

The main objective of this work was to evaluate the impact of suckling behaviour by pups on the T_b_ of their mothers. We made several *a priori* predictions concerning the patterns of variation in maternal T_b_ during lactation if it was caused by reduced capacity to dissipate heat due to being surrounded by suckling pups. First, it was predicted that T_b_ would increase during nursing bouts and the highest T_b_ of the mothers would be observed at the termination of nursing bouts (*Prediction 1*). Second, if nursing behaviour was the most important factor causing the increase in maternal T_b_ in lactation, the highest T_b_ over a day would be when mothers were nursing, and T_b_ when nursing would be higher than during other behaviours known to generate heat such as physical activity (*Prediction 2*). Third, maternal T_b_ during nursing would vary systematically with litter size, with larger litters causing the mothers to heat up more. Likewise, heavier pups later in lactation might result in more maternal hyperthermia than smaller pups earlier in lactation because the body surface in contact with pups would be greater and these pups would be hotter because they are themselves thermo-competent later in lactation (*Prediction 3*).

## Results

### *Prediction 1a*: T_b_ will increase towards the end of nursing bouts?

**Figure 1:**
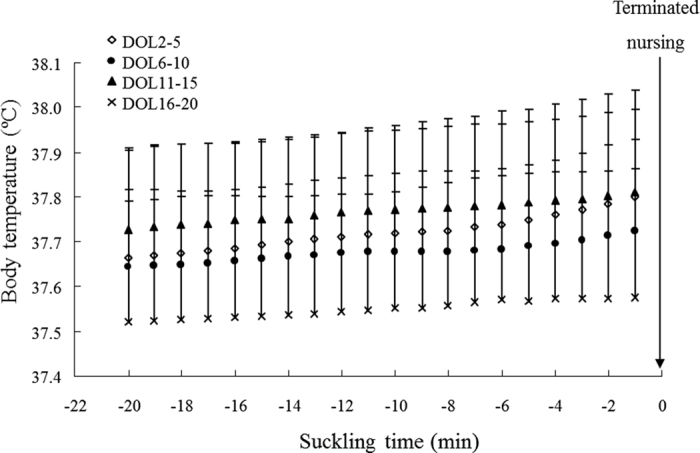
Body temperature during the last 20 minutes of nursing bouts. The data were expressed as means + S.D. for four different stages of lactation. Day of lactation (DOL) 2–5 (open-diamond), DOL 6–10 (closed-circle), DOL 11–15 (closed-triangle) and DOL 16–20 (cross). Sample sizes (n) were 18 female mice in early lactation (DOL 2–10) and 26 female mice in late lactation (DOL 11–20) although the means were obtained from 334, 420, 542 and 537 nursing bouts in DOL 2–5, DOL 6–10, DOL 11–15 and DOL 16–20, respectively.

T_b_ during four nursing bouts selected randomly from a single female mouse show the diversity of patterns of maternal T_b_ over time when females were nursing (Supplementary Fig. 1). Mean T_b_ over the last 20 minutes of all recorded nursing bouts that lasted >20 minutes, across all individuals, were compiled in four different periods of lactation (Early: DOL 2–5, Mid: DOL 6–10, Peak: DOL 11–15 and Late: DOL 16–20) (Fig. 1). Nursing T_b_ varied significantly between the four stages of lactation, but no significant differences were found with suckling time, and there was no stage by suckling time interaction (two way RM ANOVA with ID included as random factor; time, *F*_19,1660_ = 1.00, *P* = 0.46; stage of lactation, *F*_3,1660_ = 35.45, *P* < 0.001, interaction of time and stage, *F*_57,1660_ = 0.09, *P* = 1.00).

Comparing the different stages of lactation, nursing T_b_ over the last 20 minutes of suckling was significantly lower in late lactation than that in the other periods (Tukey pairwise comparisons, *P* < 0.01). In addition, nursing T_b_ was higher in early lactation and peak lactation compared to mid-lactation (Tukey pairwise comparisons, *P* < 0.05).

### *Prediction 1b*: T_b_ at the end of nursing bouts should be higher with longer nursing duration?

**Figure 2:**
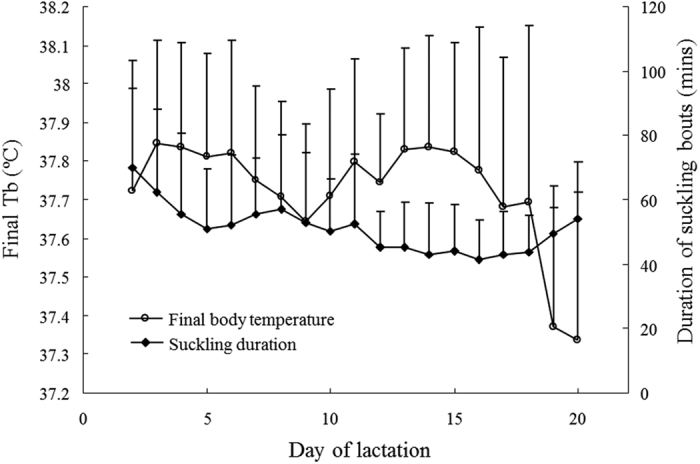
Daily means of final body temperature (T_b_) at the end of nursing bouts and duration of nursing bouts. The data are expressed as mean + S.D. (n = 26). Open-circle and closed-diamond symbols represent final T_b_ and nursing duration, respectively.

The duration (minutes) of all nursing bouts which lasted more than 20 minutes were averaged for each mouse across all nursing bouts on a given day. At the same time, mean T_b_ at termination of each nursing bout (i.e., T_b_ in the final minute, from now on called ‘final T_b_’) was also collected daily. Final T_b_ varied significantly with lactating day (Fig. 2, closed symbols, one way ANOVA, *F*_18,1814_ = 14.54, *P* < 0.001). Mean final T_b_ significantly declined on DOL 19 and 20 compared to the other days (Tukey pairwise comparison, *P* < 0.001). In addition, mean duration of nursing bouts changed with DOL (Fig. 2, open symbols, one way ANOVA, *F*_18,1814_ = 3.90, *P* < 0.001).Among days of lactation, mean duration of nursing bouts was longer on DOL 2 than that in late lactation between DOL 12 and 19 (Tukey pairwise comparison, *P* < 0.001). Mean nursing duration on DOL 3 was also longer than DOL 13, 14, 16, 17 and 18 (Tukey pairwise comparison, *P* < 0.001).

**Figure 3:**
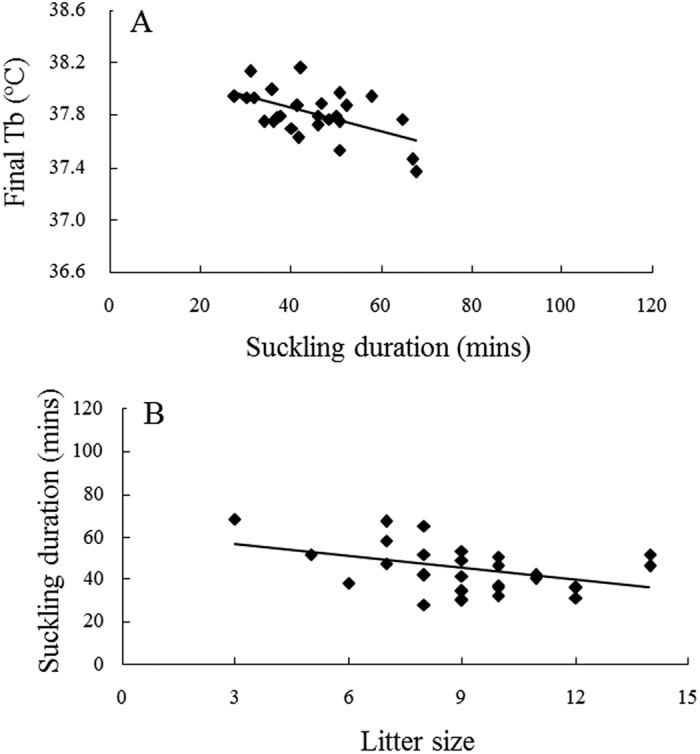
The relationships between nursing duration and final body temperature (**A**), litter size (**B**) and litter mass (**C**) at peak lactation (DOL 11–15, n = 26). Regressions are described by A: y = −0.0091x + 38.215, B: y = −0.0091x + 38.215 and C: y = −0.3793x + 71.314.

Relationships between nursing duration and final T_b_ are summarised foreach individual mouse (Table 1). Six of the 26 females showed no relationship between nursing duration and final T_b_ (*P* > 0.05), however, final T_b_ significantly decreased with longer duration of nursing bouts for the remaining females (*P* < 0.05).The same negative trend was found in relationships between mean nursing duration and mean final T_b_ when examined for the four different stages of lactation (See Fig. 3A for peak lactation). Final T_b_ was significantly, negatively related with nursing duration during early (y = −0.0074x + 38.229, *R*^2^ = 0.326, *F*_1,16_ = 7.72, *P* = 0.013), peak (Fig. 3A: y = −0.0091x + 38.215, *R*^2^ = 0.302, *F*_1,24_ = 10.38, *P* = 0.004) and late lactation (y = −0.0136x + 38.19, *R*^2^ = 0.218, *F*_1,23_ = 6.39, *P* = 0.019), but this relationship did not reach statistical significance during mid lactation, although a similar trend was visible ( y = −0.0055x + 38.012, *R*^2^ = 0.162, *F*_1,16_ = 3.09, *P* = 0.098).

In addition to stage of lactation and nursing duration, the effects of other factors such as individuals, litter size and litter mass on final T_b_ was analysed by GLM. The analysis indicated that all factors had significant influences on final T_b_ in nursing bouts (nursing duration: *F*_1,1831_ = 207.86, *P* < 0.001; individual: *F*_25,1807_ = 16.59, *P* < 0.001; litter size: *F*_1,1831_ = 139.83, *P* < 0.001; litter mass: *F*_1,1831_ = 5.77, *P* = 0.016).

Furthermore, influences of these factors on nursing duration were investigated using GLM analysis. Nursing duration was significantly related to all the parameters: individuals (*F*_25,1807_ = 14.95, *P* < 0.001), litter size (*F*_1,1831_ = 95.24, *P* < 0.001) and litter mass (*F*_1,1831_ = 100.13, *P* = 0.001). Relationships between nursing duration and litter size or litter mass were explored during the four stages of lactation (Fig. 3B,C respectively show the relationships at peak lactation). Nursing duration was significantly longer in mothers with smaller litters at all four stages of lactation (Early: y = −3.8651x + 92.239, *R*^2^ = 0.347, *F*_1,16_ = 8.51, *P* = 0.010; Mid: y = −3.1911x + 79.674, *R*^2^ = 0.321, *F*_1,16_ = 7.56, *P* = 0.014; Peak, Fig. 3B: y = −1.8323x + 61.627, *R*^2^ = 0.171, *F*_1,24_ = 4.96, *P* = 0.036; Late: y = −1.9357x + 62.526, *R*^2^ = 0.259, *F*_1,23_ = 8.05, *P* = 0.009).

Nursing duration was also negatively related to litter mass at all stages of lactation (Early: y = −1.96x + 107.3, *R*^2^ = 0.511, *F*_1,16_ = 16.7, *P* < 0.001; Mid: y = −0.8172x + 89.805, *R*^2^ = 0.382, *F*_1,16_ = 9.75, *P* = 0.007; Peak, Fig. 3C: y = −0.3793x + 71.314, *R*^2^ = 0.252, *F*_1,24_ = 8.09, *P* = 0.009) except late lactation where it marginally failed to reach significance (Late: y = −0.1896x + 61.208, *R*^2^ = 0.138, *F*_1,23_ = 3.69, *P* = 0.067). Nursing duration was shorter for mothers with bigger or heavier litters.

### *Prediction 1c*: Mean T_b_ while nursing inside nest should be higher when total nursing time is longer?

**Figure 4:**
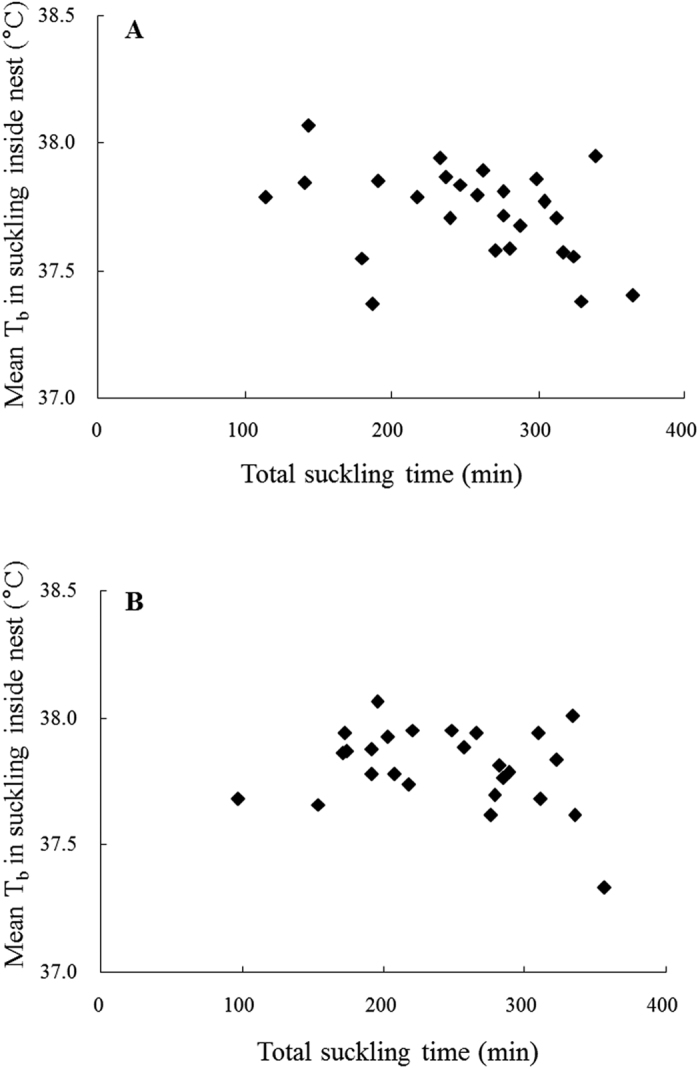
Relationship between mean nursing T_b_ and total time spent nursing inside the nest (n = 26). (**A**) represents means over the whole lactation (DOL 2–20), and (**B**) represents means over the peak lactation (DOL 11–15). The regressions are described by y = −0.001x + 37.973 (**A**) and y = −0.0006x + 37.946 (**B**) and were not significant p > 0.05.

The relationship between total nursing time during the 8-hour observation and mean T_b_ during nursing inside the nest was examined between individual mice across the whole lactation and at peak lactation (Fig. 4). It was predicted that the more time mice spent nursing inside the nest during behavioural observations, the hotter nursing T_b_ they would exhibit. However, there was no significant relationship between mean nursing T_b_ and total time during nursing inside the nest when considering both the entire lactation (Fig. 4A: y = −0.001x + 37.973, *R*^2^ = 0.122, *F*_1,24_ = 3.42, *P* = 0.077) or the peak lactation (Fig. 4B: y = −0.0006x + 37.946, *R*^2^ = 0.060, *F*_1,24_ = 1.54, *P* = 0.227).

### *Prediction 2a*: Nursing inside nest should generate the hottest T_b_?

**Figure 5:**
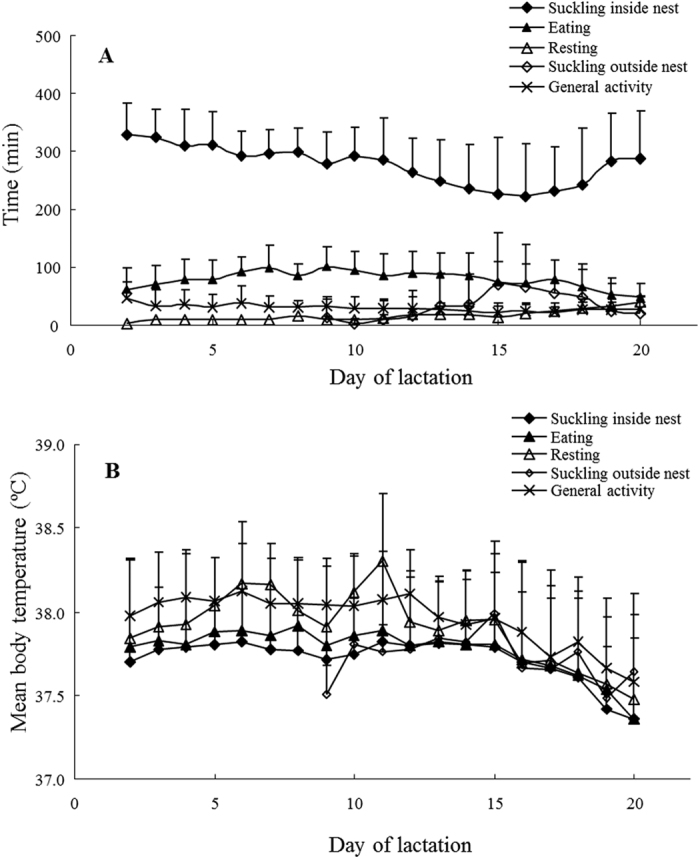
Time spent on various behaviours and their relationship to body temperature. (**A**) shows the total time spent on different behaviours during the behavioural observation in lactation and (**B**) shows the daily body temperatures associated with different behaviours. Behaviours are categorised as nursing inside nest (closed-diamond), eating (closed-triangle), resting (open-triangle), nursing outside nest (closed-diamond) and general activity (cross). All data are shown as means + S.D. Sample sizes (n) vary from 18 to 26 mice depending on days of lactation (refer to the details in Supplementary Table 1).

Mean time spent on different behaviours was calculated on a daily basis across the 26 females. Of the major behaviours in lactating mice, nursing inside nest was a dominant activity during observation hours in daytime (Fig. 5A). Mean time spent on nursing inside nest varied with DOL (one way ANOVA, *F*_18,384_ = 4.94, *P* < 0.001) (Fig. 5A, closed diamonds). Time spent on nursing inside the nest declined significantly from 328.8 ± 53.1 minutes (71.2 ± 10.1% of the 8-hour observation) on DOL 2 to 220.8 ± 91.7 minutes (50.7 ± 19.3%) on DOL 16 (Tukey pairwise comparisons, *P* < 0.01). In terms of time, eating behaviour was the second most frequent behaviour (Fig. 5A, closed triangles). During the observation hours, eating time also varied with DOL (one way ANOVA, *F*_18,384_ = 3.94, *P* < 0.001) (Fig. 5A, closed triangles). However, its percentage ranged between 10.5 ± 5.5% on DOL 20 and 21.9 ± 7.8% on DOL 9 which was notably smaller than the time spent on nursing inside the nest. In contrast, suckling mice started leaving their nest from DOL 9 and significantly increased time spent on nursing outside the nest as lactation progressed (one way ANOVA, *F*_18,384_ = 6.18, *P* < 0.001, Fig. 5A, open diamonds). Time spent on nursing outside nest was 13.7 ± 30.1 minutes (2.9 ± 6.3%) on DOL 9 and increased to 68.7 ± 90.3 minutes (17.3 ± 23.1%) on DOL 15 and 66.0 ± 73.9 minutes (15.30 ± 17.68%) on DOL 16 (Tukey pairwise comparisons, *P* < 0.05).

In addition, the total percent nursing time (time spent nursing inside + time spent nursing outside the nest) increased to 69.0 ± 10.8% on DOL 15 and 66.0 ± 9.5% on DOL 16. The total nursing time did not vary significantly with DOL (one way ANOVA *F*_18,384_ = 1.55, *P* = 0.07). Therefore, the proportion of the nursing time to feed offspring was constant throughout lactation, although nest attendance was significantly reduced in late lactation.

Time spent on resting gradually elevated with advanced DOL (one way ANOVA, *F*_18,384_ = 3.27, *P* < 0.001) (Fig. 5A, open triangles) and maximised at 39.6 ± 39.3 minutes (8.75 ± 8.78%) on DOL 20.

T_b_ while nursing inside the nest significantly declined as lactation advanced (one way ANOVA, *F*_18,383_ = 5.86, *P* < 0.001) (Fig. 5B, closed diamonds). Significant differences in daily changes in nursing T_b_ were found only between DOL 2 and DOL 19 or 20 (Tukey pairwise comparisons, *P* < 0.05). T_b_ varied significantly with behavioural type (nursing inside nest, eating, resting, nursing outside nest and general activity) and DOL (Fig. 5B, two way ANOVA; behavioural type, *F*_4,1481_ = 30.82, *P* < 0.001; DOL, *F*_18,1481_ = 16.51, *P* < 0.001; interaction, *F*_65,1481_ = 0.79, *P* = 0.88). T_b_ when nursing inside nest was lower than that when resting and during general activity (Tukey pairwise comparisons, *P* < 0.01). In addition, resting T_b_ in lactation gave as high a T_b_ as the other behaviours (Tukey pairwise comparisons, *P* > 0.05).

### *Prediction 2b*: The highest T_b_ should be found during nursing bouts?

**Figure 6:**
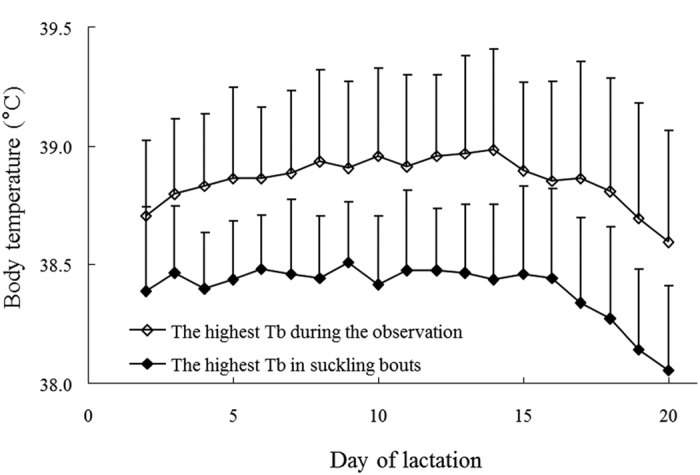
Comparison of highest T_b_ found in nursing bouts and entire observation hours. All data are shown as means + S.D. Sample sizes (n) vary from 21 to 26 depending on days of lactation (refer to the details in Supplementary Table 1). Open-diamond represents the highest body temperature observed during the 8-hour behavioural observation between 10.00 h and 18.00 h. Closed-diamond represents the highest body temperature observed in nursing bouts between 10.00 h and 18.00 h.

The highest T_b_ measured in the observation period was compared to the highest T_b_ recorded when nursing inside nest through DOL 2–20 (Fig. 6). The highest T_b_ measured was 38.98 ± 0.43 °C on DOL 14 compared to a maximal T_b_ of 38.48 ± 0.26 °C during nursing bouts on DOL 12. The difference between the maximal temperatures measured during the whole observation period or nursing bouts only increased as DOL advanced (DOL, *F*_18,764_ = 3.66, *P* < 0.001) although there were no differences between the two highest values in the whole observation and in nursing bouts in early lactation (DOL 2–7) (Tukey pairwise comparisons, *P* > 0.05). From DOL 8 to DOL 20 except DOL 9, the highest temperatures in the observation hours were significantly higher than those in nursing bouts (Tukey pairwise comparisons, *P* < 0.05).

### *Prediction 3:* Litter size and litter mass will be positively related to nursing T_b_.

**Figure 7:**
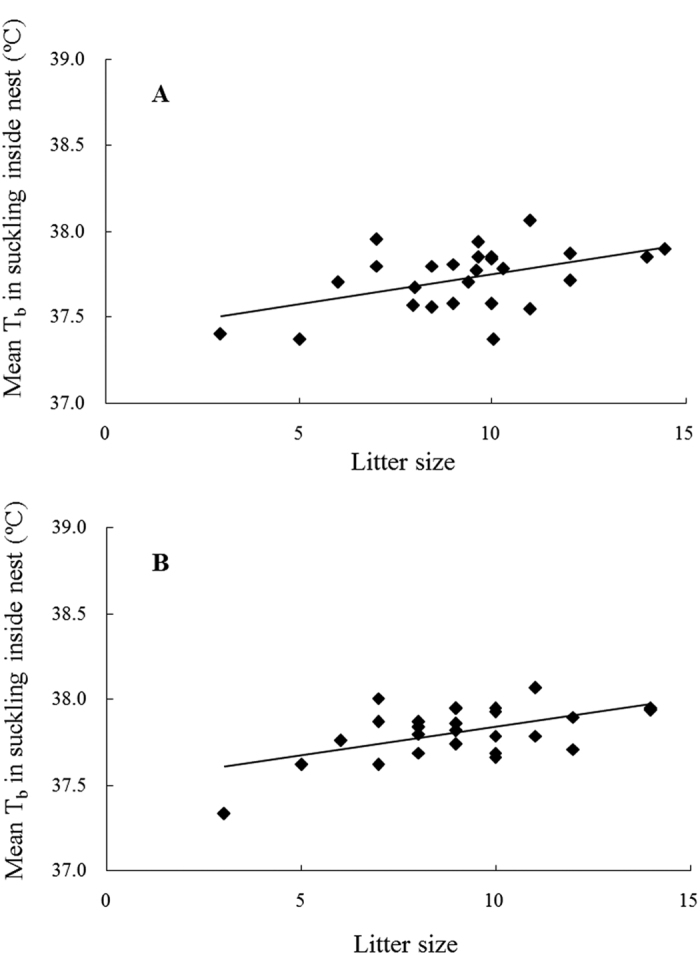
The relation between litter size and maternal body temperature during nursing inside nest. (**A**) represents means over the whole lactation (DOL 2–20) (y = 0.0344x + 37.403, *R*^2^ = 0.225, *F*_1,24_ = 6.988, P = 0.014), and (**B**) represents means over the period of peak lactation (DOL 11–15) (y = 0.0335x + 37.502, *R*^2^ = 0.296, *F*_1,24_ = 10.09, *P* = 0.004).

T_b_ while nursing inside the nest of individual mice was averaged over the whole lactation or peak lactation. Nursing T_b_ over the whole of lactation varied from 37.37 °C to 38.07 °C and was significantly, positively related to litter size (Fig. 7A: y = 0.0344x + 37.403, *R*^*2*^ = 0.225, *F*_1,24_ = 6.988, *P* = 0.014). At peak lactation, nursing T_b_ ranged between 37.33 °C and 38.06 °C and was also significantly related to litter size (Fig. 7B: y = 0.0335x + 37.502, *R*^*2*^ = 0.296, *F*_1,24_ = 10.09, *P* = 0.004).

**Figure 8:**
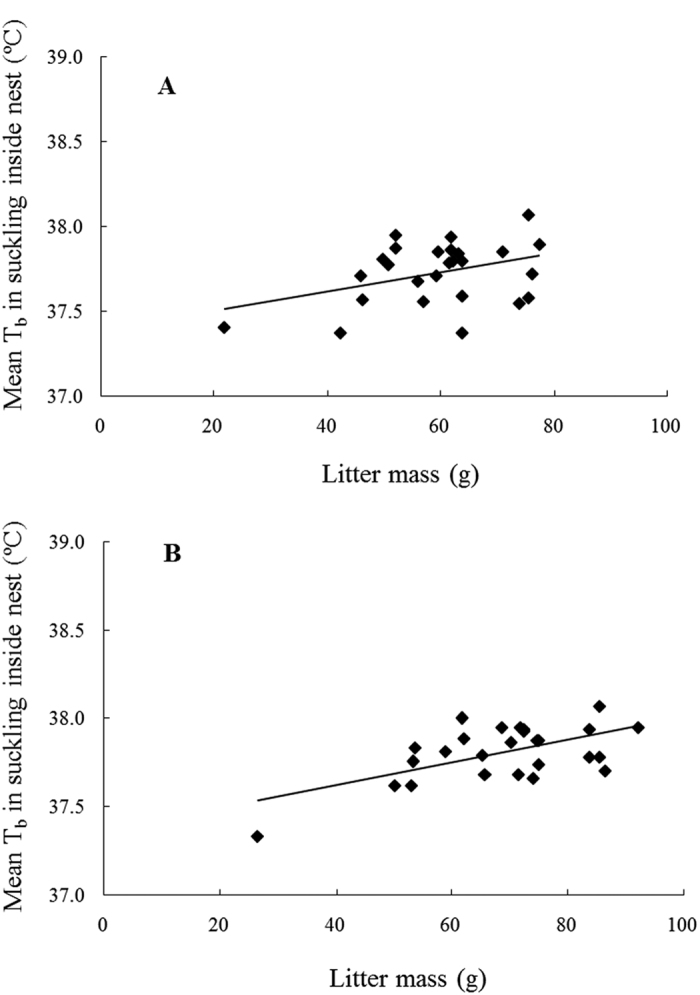
The effect of litter mass on maternal body temperature during nursing inside nest. (**A**) represents means over the whole lactation (DOL 2–20) (y = 0.0056x + 37.39, *R*^2^ = 0.1486, *F*_1,24_ = 4.166, P = 0.052), and (**B**) represents means over the peak lactation (DOL 11–15) (y = 0.0064x + 37.366, *R*^2^ = 0.347, *F*_1,24_ = 12.66, *P* = 0.002). The sample sizes (n) are 26 mice in the both figures. When the smallest litter size (=3) was removed (n = 25 mice), the regressions changed to A: y = 0.0049x + 37.465, *R*^2^ = 0.065, *F*_1,23_ = 1.603, *P* = 0.218 and B: y = 0.0036x + 37.572, R^2^ = 0.065, F_1,23_ = 2.939, P = 0.1.

Litter mass varied from 21.73 g to 75.53 g on average over the whole lactation and from 26.61 g to 75.07 g at peak lactation. The positive relationship between nursing T_b_ and litter mass marginally failed to reach significance when taken as a mean over the whole period of lactation (Fig. 8A: y = 0.0056x + 37.39, *R*^*2*^ = 0.1486, *F*_1,24_ = 4.166, *P* = 0.052), but there was a significant positive relationship between nursing T_b_ and litter mass at peak lactation (Fig. 8B: y = 0.0064x + 37.366, *R*^*2*^ = 0.347, *F*_1,24_ = 12.66, *P* = 0.002).

### Do eating behaviour and food intake affect elevation in T_b_?

**Figure 9:**
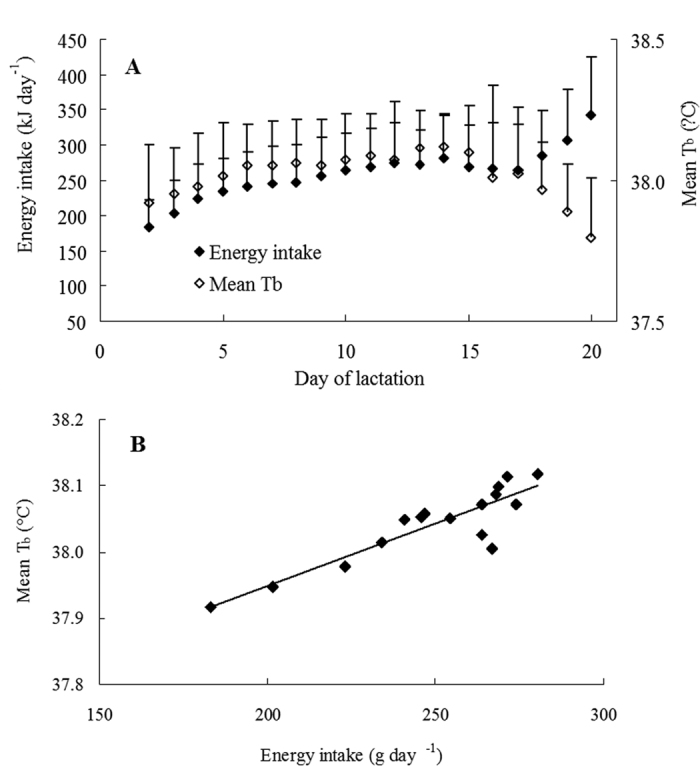
The relationship between mean energy intake and mean T_b_. (**A**) shows daily changes of energy intake and mean T_b_ averaged across 26 females during lactation. (**B**) shows the relationship between mean energy intake and mean T_b_ between DOL 2 and DOL 17 (n = 16 days). The regression is described by y = 0.0254x + 37.574.

Nursing T_b_ was influenced by litter size and litter mass. However, nursing T_b_ did not differ from mean T_b_ related to other behaviours. There might be more important parameters associated with thermoregulation during lactation. First, the effect of energy intake on T_b_ was investigated as heat is generated through the process of food digestion. Figure 9A shows the daily changes in mean energy intake and mean T_b_ during lactation across the 26 individuals. Maternal energy intake and mean T_b_ varied in parallel from DOL 2 until DOL 17. Energy intake increased from DOL 18 onwards due to additional food consumption by growing young. Fluctuations in mean T_b_ was thus compared with the increase in mean energy intake from DOL 2 until DOL 17 (Fig. 9B). Up to DOL 17, both energy intake and mean T_b_ were maximised on DOL 14. Mean energy intake varied from 183.01 ± 37.91 kJ day^−1^ on DOL 2 to 280.55 ± 62.08 kJ day^−1^ on DOL 14. Mean T_b_ increased from 37.92 ± 0.21 °C on DOL 2 to 38.12 ± 0.12 °C on DOL 14. Mean T_b_ was significantly related to mean energy intake during this period of lactation (DOL 2–17) (Fig. 9B: y = 0.0254x + 37.574, *R*^*2*^ = 0.79, *F*_1,14_ = 53.74, *P* < 0.001).

Mean T_b_ when eating might be related to feeding time and to test this total time spent on eating and mean T_b_ when eating at peak lactation (DOL 11–15) were compared. Lactating mice spent from 33.0 minutes (6.9% of the 8-hour observation) to 166.8 minutes (34.8%) on feeding during the observation hours at peak lactation (n = 26). Mean T_b_ when eating ranged between 37.51 °C and 38.30 °C (n = 26). There was no significant relationship between eating time and T_b_ at peak lactation (y = 0.0018x + 37.657, *R*^*2*^ = 0.0998 *F*_1,24_ = 1.17, *P* = 0.29).

## Discussion

The heat dissipation hypothesis predicts that the risk of hypothermia during lactation may limit performance. Lactating rodents show a disruption of their T_b_ rhythms. This is mainly seen as a decrease in the amplitude of the rhythm that is primarily caused by an increase in light phase T_b_ to levels similar to normal dark phase T_b_ (*Mus musculus*^18^, *Rattus norvegicus*^22^*, Phodopus sungorus and Phodopus campbelli*^26^). During the light phase, female mice spend most of their time in their nest with their offspring^20^. Here we tested the hypothesis that the observed hyperthermia is caused by suckling offspring surrounding the female thus limiting her capacity to dissipate heat. If confirmed this would indicate that heat dissipation limits are likely a phenomenon specific to lactation.

We tested three predictions based on the hypothesis. First, if suckling offspring limit the capacity of the female to dissipate heat, we predicted T_b_ would increase with the duration of nursing bouts and that T_b_ would reach its maximum at the end of the nursing bout. Contrary to this expectation, nursing T_b_ did not increase within nursing bouts and final T_b_ was not positively, but negatively related to nursing bout duration. These results are in agreement with some observations made previously in rats (*Rattus norvegicus*) where in approximately 60% of nursing bouts, higher T_b_ was recorded during the bouts rather than at the end of the bouts^25^, and contradict the idea that females prolong their bouts until they are at near fatal hyperthermia. An association between T_b_ and nest attendance has been shown in Dwarf hamsters, where females showed increasing T_b_ while in the nest and a decrease in T_b_ upon leaving the nest^20^. Also, in female rats increased duration of suckling bouts were found when females were exposed to cold vs. warmed pups or when mothers were placed in a room with a cold vs. warm ambient temperature^22^. These observations contradict our results. The differences between our study and these previous studies could reflect real biological differences between the species under investigation. For example, rats are much larger than mice and hence have a lower surface to volume ratio that may make heat dissipation more difficult. Furthermore dwarf hamsters may have better fur insulation than mice also preventing efficient dissipation of heat. On the other hand the differences may reflect methodological differences between studies. In both previous studies transmitters were used to determine the location of the females inside or outside of a nest box and no direct observation of suckling behaviour were made and neither study tested whether there was a consistent rise in T_b_ over nursing bouts. Our study clearly showed that the highest T_b_ were not observed while the females were nurturing their offspring inside the nest and that T_b_ did not increase throughout the nursing bouts as would be expected if suckling offspring were causing female hyperthermia.

During the last few days of lactation, decreases in maternal T_b_ when nursing inside nest as well as final T_b_ at the end of nursing bouts were observed, which is consistent with a decrease in mean T_b_ at the end of lactation^18^. Dwarf hamsters (*P. sungorus* and *P. campbelli*) also declined T_b_ during the light phase at the later stages of lactation^20^. At this stage of lactation, the number of offspring surrounding the mothers reduced as they became more mobile and able to obtain solid food by themselves. This might be the reason why T_b_ in nursing bouts was significantly lower in the last stages of lactation compared to earlier stages of lactation since litter size was significantly related to maternal T_b_ during nursing inside nest.

The second prediction tested was that maximum T_b_ over a day should occur during nursing, and T_b_ should be higher when nursing than during other behaviours known to generate heat. In the current experiment, the maximal T_b_ measured during the 8 h observation period did not occur while females were nursing, but during other behaviours. On average over the course of lactation the highest T_b_ were measured while females engaged in physical activity. These results again contradict the idea that females prolong their nursing bouts until they reach near fatal hyperthermia. Furthermore, maximal nursing T_b_ measured in the present study was lower than the rectal temperature of mice exposed to 34 °C for an hour in the study by Harikai *et al*.^27^ although the measuring sites of T_b_ were different between the two studies. Since rectal temperature is lower than T_b_ measured in gut^28^, T_b_ increased more under the hot condition than in lactation in mice. In the report by Harikai *et al*.^27^, mice reduced food consumption, however, lactating mice in this experiment combined hyperthermia with high levels of energy intake. During heat exposure reducing food intake may be a viable option but lactating mice could only do this if they sacrificed milk production which may explain their different responses.

Also, the thermal set point might be elevated in lactating mice since heat production of lactating and non-lactating rats were similar in the cold at 4 °C although only lactating rats could maintain their core T_b_^29^. Similarly, lactating mice in the cold (8 °C) and warm (21 °C) showed no difference in their resting metabolic rate (RMR)^4^. Mass and activities of brown adipose tissue (BAT) related to non-shivering thermogenesis is decreased in lactating female mice^30^,^31^,^32^.

MF1 mice spent less time on nursing inside the nest on DOL 16 compared to the beginning of lactation, but did not change total contact time (i.e., nursing inside and outside the nest) with their offspring through lactation. In rats, nesting time with offspring was considerably higher on DOL 2 and progressively decreased as lactation advanced^22^. A similar reduction of mother-young contact time with day of lactation was found in Dwarf hamsters^20^. In addition, nursing duration was relatively brief when room temperature was increased or mother rats were warmed^22^. These results imply that peak lactation is the period where the risk of hyperthermia is the greatest for lactating rodents. Although, the fact that maximal T_b_ did not occur during nursing bouts indicates that offspring huddling around the mother are not the main cause of the hyperthermia at peak lactation.

Lactating mice were hotter when moving around or nest-building than any other behaviour, including nursing, eating and resting. This is consistent with the concept that locomotor activity contributes to T_b_ change in non-reproductive rodents^33^,^34^ and indicates that it may also influence T_b_ in lactating mice. However, activity levels were considerably reduced throughout lactation^18^ and locomotor activity is unlikely to cause the prolonged maternal hyperthermia observed in lactating mice.

Another important factor that can cause an increase in T_b_ is digestion of food. No significant relationship between the time spent eating and T_b_ was found. The behavioural observations were conducted during the light phase though, while mice predominantly feed at night. When investigating the relationship between total energy intake and mean T_b_ over the day a significant positive relationship was found. Therefore, the intensive feeding during lactation and associated acute increase in metabolic heat production may lead to elevation of T_b_ at the peak lactation.

Mothers raising bigger or larger litters were expected to have increased nursing T_b_ according to our third prediction. Indeed, a positive relationship between litter size or litter mass and maternal T_b_ while nursing inside the nest was found throughout lactation. This is consistent with the finding that rats raising large litters spent less time on nursing than rats raising small litters^22^ and a similar positive relationship between litter size and T_b_ was also found in Dwarf hamsters, but only on lactation day 12 and 17^20^. These results imply that larger litter size induced a hotter environment for mothers than smaller litter size due to pups limiting the mother’s heat dissipating capacity. The fact that nest attendance time decreased in warmed mother rats compared with unmanipulated mothers also agrees with this interpretation^22^. If this were the case, one would expect a consistent increase in T_b_ with nursing duration, which was not observed in the current study. Also, the positive relationship between litter size and litter mass is driven mainly by the smallest litter and when this litter is removed from analysis, no significant relationship remains. An alternative explanation may be that milk stored in the mammary glands was emptied faster from mothers with larger litter sizes resulting in shorter nursing bouts.

Lactating mice show a pronounced increase in energy intake over the course of lactation until a plateau is reached^1^. This increase in energy intake seemed to parallel changes in T_b_ and a significant positive relationship between energy intake and T_b_ was found. Metabolic heat generated from food digestion may thus be an important factor determining female T_b_ during lactation. In contrast, food deprivation did not affect core T_b_ during lactation^29^. Another important factor causing hyperthermia in lactation may be heat generated as a by-product of milk synthesis. Daily growth of litters significantly synchronised with daily changes in maternal T_b_^18^. This might suggest that milk production contributes to heat generation when growth of the litter is taken into account, as milk delivery is positively related to litter mass^1^,^6^,^35^. On the other hand, milk production had no effects on core T_b_ in mother rats when litter size was adjusted to eight for all litters^29^. In the current study, litter size widely varied from 3 to 14 at weaning, which might have precipitated the significant relationship between maternal T_b_ and litter mass.

## Conclusion

Nursing behaviour could be a main cause of hyperthermia in lactating mice if nursing offspring significantly enhanced heat retention, and therefore, prevented mothers from dissipating heat. However, our observations of T_b_ changes while engaging in nursing and other behaviours, showed that T_b_ did not increase with nursing duration and that the maximum T_b_ experienced by the mother did not occur during nursing bouts. Nursing offspring and/or nursing behaviour were thus not the crucial factors for maternal hyperthermia, pointing towards a role for heat generation induced by food digestion and/or milk production in causing hyperthermia in lactating mice. Indeed, energy intake and litter mass, which is known to be related to milk energy output, were significantly related to an increase in mean T_b_. These data indicate that heat dissipation limits are probably not a phenomenon specific to lactation but more generally applicable across all conditions.

## Methods

### Behavioural observations

Behavioural observations were conducted using the same female mice (outbred MF1: *Mus musculus*; Harlan UK Ltd, Bicester, UK) measured in experiments described in Gamo *et al*.^17^,^18^. Mice were implanted with passive transmitters (Vital view) reporting their T_b_ and physical activity levels at 1 minute intervals. In 2005, individual female mice were observed in the second half of lactation from day 11 to day 20 (date of lactation (DOL) 11 to DOL 20) (n = 8 litters). The observation period was extended to 19 days from DOL 2 until DOL 20 in 2006 (n = 8 litters) and 2007 (n = 10 litters). One transmitter failed midway through lactation and we discontinued observations on this individual after that date. Behaviour monitoring was performed for eight hours a day between 10.00 h and 18.00 h, in the light phase, across all three years. To compare the results from the behaviour observations with the T_b_ recorded by the VitalView system, animals were visually monitored every minute by direct observation. Behaviours were logged each minute and classified as nursing inside nest, nursing outside nest, eating, grooming, drinking, resting and general activity. A nest was defined as any place where most of pups were located within paper bedding. When the majority of pups were attached to their mothers either inside or outside their nest, maternal behaviour was recorded as nursing. In late lactation, it was occasionally observed that mothers ate food from the hoppers with one or two pups attached. In this case, their behaviour was regarded as eating. Likewise, the effect of a small number of pups (<3) attached to their mothers was ignored when their mothers were pre-dominantly grooming, drinking or moving around. By contrast, resting behaviour was recorded when mothers were sleeping, without any pups attached, either inside or outside their nest.

Food intake was calculated by subtracting the amount of food in the hoppers from that of the previous day. Energy intake was then estimated using estimates of dry mass content and apparent digestibility determined previously in lactating MF1 mice (for a full description of the methods see Gamo *et al*.^17^,^18^).

All procedures concerning animal care and treatment were carried out in accordance with the protocols approved by the ethical committee for the use of experimental animals of the University of Aberdeen, and were licensed by the UK Home Office under PPL 60/3705.

### Data collection

T_b_ and activity counts were correlated with observed behaviours by co-ordinating the recording times. Therefore, for every minute of the 8-hour observation period one of the behavioural categories, with T_b_ and activity counts was recorded. Uninterrupted nursing bouts lasting longer than 20 minutes were included in the analysis of temperature trends. A total 1,833 nursing bouts were collected across the 26 females over the 19 days of lactation (DOL 2–20). The sample sizes varied depending on day of lactation (Supplementary Table 1).

Total time (minutes) spent nursing was calculated over the whole 8-hour observation period regardless of the nursing bout durations (including bouts <20 min). Mean T_b_ referring to nursing inside nest was also calculated from all data recorded as nursing inside nest. Likewise, total times (minutes) spent on other behaviours such as eating, resting, nursing outside nest and general activity were calculated. Mean T_b_ corresponding to each identified behaviour were calculated across all observations. Total time and mean T_b_ corresponding to all behaviours were averaged daily across 17 female mice during DOL 2–10 and across 25 female mice during DOL 11–20.

### Data analysis

All data are expressed as means + standard deviation (S.D.). *Prediction 1a:* In the analysis only nursing bouts of 20 min and longer were included. Mean Tb was calculated for each nursing bout over the last 20 min of the bout (i.e., suckling time −20 to −1) and compiled for the four stage of lactation (Early: DOL 2–5, Mid: DOL 6–10, Peak: DOL 11–15 and Late: DOL 16–20). Analysis of variance (ANOVA) was used for assessing significant differences in T_b_ with suckling time or stage of lactation. In this analysis, mother ID was included as a random factor to correct for repeated measures. Furthermore, Tukey post-hoc tests were conducted to compare the different stages of lactation. *Prediction 1b:* Daily mean duration of nursing bouts was calculated for all nursing bouts lasting 20 min or more (i.e, nursing duration). In addition, daily means for T_b_ at the termination of these nursing bout (i.e., T_b_ in the final minute of a nursing bout, from now on called ‘Final T_b_’) was calculated. One way ANOVA’s were used to test for differences in duration of nursing bouts or Final T_b_ with DOL. In these analyses, mother ID was included as a random factor to correct for repeated measures. Linear regressions were used to investigate relationships between nursing duration, final T_b_, litter mass and litter size during the different stages of lactation. In addition, generalised linear models (GLM) were run to investigate of several predictors, i.e., DOL, individuals, litter size and litter mass on final T_b_ and nursing duration. *Prediction 1c:* Daily means of total duration of nursing during the 8 h observation period and mean T_b_ while nursing inside the nest were calculated for each individual mother over the whole of lactation or peak lactation. Linear regressions were then applied to test whether there was a relationship between these two variables. *Prediction 2a:* The time spent on different behaviours, i.e., nursing inside the nest, nursing outside the nest, eating, resting and general activity, was calculated as daily averages for each of the individual mothers. Total time spent on different types of behaviours was also compared with days of lactation by ANOVA followed by Tukey post-hoc tests for each behaviour seperately. In addition, mean T_b_ recorded during the different behaviours were analysed by two-way ANOVA with DOL and behavioural type as fixed factors. Again mother ID was included in these models to account for repeated measures. *Prediction 2b*: The highest recorded values of T_b_ during the whole observation period was compared to the highest T_b_ recorded during nursing bouts inside the nest for each day of lactation using one way ANOVA with day of lactation as fixed factor and ID as random factor. Prediction 3: Linear regressions were used to examine the relationships between mean nursing T_b_ and litter size or litter mass. These analysis were performed for mean nursing T_b_ calculated over the whole period of lactation and nursing T_b_ during peak lactation separately.

Statistical analyses were carried out using the R programme (R Development Core Team, 2007) and SPSS.

## Additional Information

**How to cite this article**: Gamo, Y. *et al*. Limits to sustained energy intake XXIV: impact of suckling behaviour on the body temperatures of lactating female mice. *Sci. Rep.* **6**, 25665; doi: 10.1038/srep25665 (2016).

## Supplementary Material

Supplementary Information

## Figures and Tables

**Table 1:** Relationships between duration of nursing bouts and final T_b_ at the end of nursing bouts.

Mouse ID	Litter size	n	Slope	Intercept	*R*^*2*^	d.f.	*F*	
1	10	16	−0.0135	38.492	0.156	1,14	2.596	
2	10	32	−0.0140	38.055	0.263	1,30	10.69	*
3	12	46	−0.0085	38.21	0.13	1,44	6.583	
4	11	23	−0.0197	38.901	0.221	1,21	5.959	
5	9	33	−0.0119	38.203	0.143	1,31	5.188	
6	9	46	−0.0110	38.18	0.206	1,44	11.43	*
7	14	25	−0.0037	37.871	0.045	1,23	1.072	
8	11	29	−0.0137	38.212	0.25	1,27	8.978	
9	10	88	−0.0096	37.969	0.194	1,86	20.70	*
10	8	126	−0.0053	37.708	0.042	1,124	5.455	
11	9	97	−0.0090	38.072	0.207	1,95	24.75	*
12	6	100	−0.0068	38.033	0.189	1,98	22.82	*
13	8	78	−0.0167	38.397	0.112	1,76	9.619	
14	10	65	−0.010	38.209	0.189	1,63	14.68	*
15	12	96	−0.016	38.53	0.24	1,94	29.7	*
16	5	82	−0.0025	37.47	0.05	1,80	4.193	
17	9	88	−0.0039	37.89	0.054	1,86	4.901	
18	3	89	−0.0019	37.485	0.04	1,87	3.638	
19	8	72	−0.001	37.697	0.012	1,70	0.821	
20	7	97	−0.004	38.157	0.111	1,95	11.84	*
21	10	103	−0.0051	37.979	0.065	1,101	7.042	
22	14	79	−0.0034	38.111	0.091	1,77	7.737	
23	8	91	−0.0052	37.926	0.157	1,89	16.59	*
24	7	76	−0.0062	38.102	0.132	1,74	11.27	*
25	7	81	−0.0021	37.681	0.023	1,79	1.885	
26	9	75	−0.0016	37.993	0.015	1,73	1.101	

Using linear regression, slopes, intercepts and R-squared of the relationship between duration of nursing bouts (x-axis) and final T_b_ at the end of nursing bouts (y-axis) were determined on individual mice. Litter size shows the number of offspring at weaning. Sample size (n) shows the number of nursing bouts recorded during lactation. Degrees of freedom (d.f.), F values for each individual are given in the table.

^*^Indicates significance at the p < 0.05 level using the Holm-Bonferroni sequential adjustment for multiple testing.
