# Supplementary material for: Limits to sustained energy intake XXIV: impact of suckling behaviour on the body temperatures of lactating female mice
Source: Sci Rep. 2016 May 9;6:25665. doi: 10.1038/srep25665 (PMC4860708; doi:10.1038/srep25665)
Supplement: Supplementary Information [file srep25665-s1.pdf]

Limits to sustained energy intake XXIV: impact of suckling behaviour on the body temperatures of lactating female mice

Gamo, Y., Bernard, A., Troup, C. , Munro, F. , Derrer, K., Jeannesson, N., Campbell, A., Gray, H., Miller, J., Dixon, J., Mitchell, S.E., Hambly, C., Vaanholt, L.M., and Speakman, J.R.

Supplementary Fig. 1

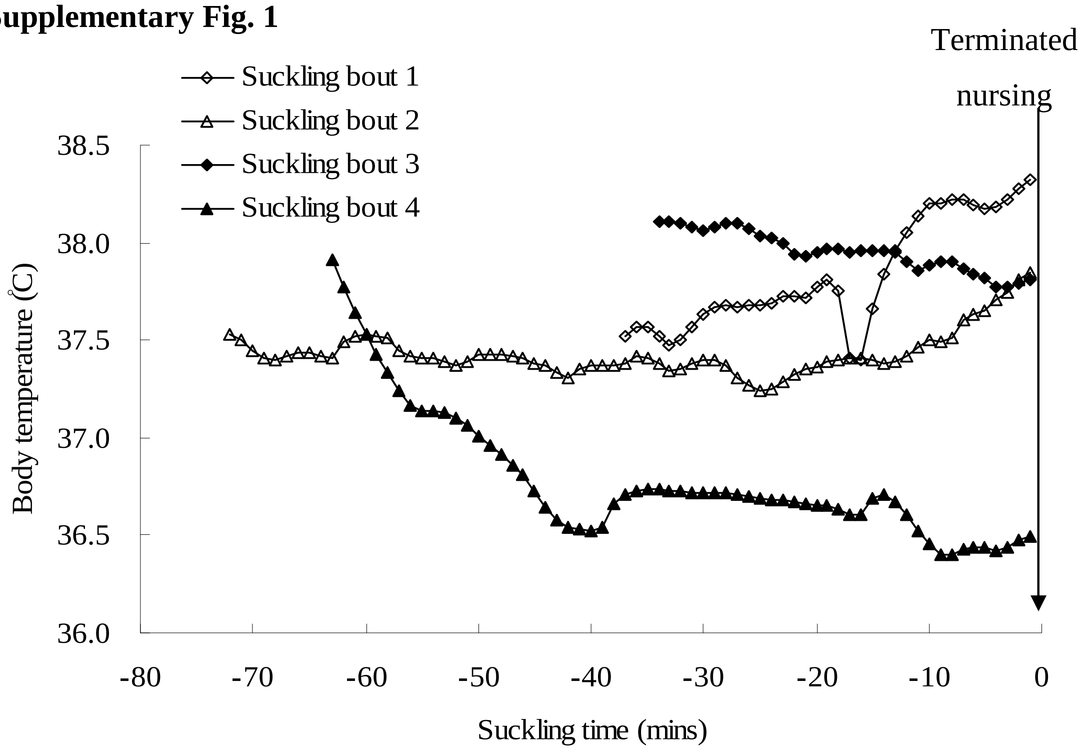

Body temperature development during nursing bouts. The data show changes of body temperatures in four randomly selected nursing bouts. Each symbol represents body temperature in a single bout. Time 0 means termination of nursing.

Supplementary Table 1. Summary of the number of observed subjects and collected nursing bouts during the 8-hour behavioural observation in lactation.

Day of lactation	The number of observed mice	The number of nursing bouts
2	18	79
3	18	78
4	18	85
5	18	92
6	18	83
7	18	81
8	18	85
9	18	80
10	18	91
11	21	100
12	23	107
13	26	119
14	26	115
15	25	101
16	25	105
17	25	111
18	25	109
19	23	116
20	21	96
